# Contributing to agricultural mix:analysis of the living standard measurement study – Integrated survey on agriculture data set

**DOI:** 10.1016/j.dib.2018.07.057

**Published:** 2018-07-27

**Authors:** Romanus Osabohien

**Affiliations:** Department of Economics and Development Studies, Covenant University, Ota, Nigeria

**Keywords:** GHS, LSMS-ISA, Agriculture

## Abstract

The Living Standard Measurement Study- Integrated Survey on Agriculture (LSMS-ISA) is a General Household Survey (GHS) and a cross-sectional survey consisting of 22,000 households which is carried out periodically across the globe. Currently, the GHS has three panels consisting of 5000 households of the GHS collecting additional data on agricultural activities, other household income activities, and household expenditure and consumption, among others. This is to improve data from the agricultural sector and the linkage to other facets of households’ characteristics and outcomes. The LSMS data-set, questionnaire, and basic information document are freely available online at: http://microdata.worldbank.org/index.php/catalog/2734.

**Specifications Table**

**Table:** 

Subject area	Economics, Agriculture
More specific subject area	Microeconomics; agricultural economics
Type of data	Primary/survey data conducted twice a year for two farming seasons (post-planting and post-harvest season
How data was acquired	Data was collected through a survey. For Nigeria, the GHS-Panel sample^1^ is fully integrated with the 2010 GHS Sample. The GHS sample is comprised of 60 Primary Sampling Units (PSUs) or Enumeration Areas (EAs) chosen from each of the 36 States and the Federal Capital Territory (FCT, Abuja) in Nigeria. This results in a total of 2220 EAs nationally. Each EA constitutes 10 households in the GHS sample, resulting in a sample size of 22,200 households.
Data format	Survey data obtained raw from LSMS-ISA
Experimental factors	For this experiment, two versions of the household questionnaire were administered. The GHS-Panel Wave 3 was administered in two visits: post-planting (September–November 2015) and post-harvest (February–April 2016).
Experimental features	The tracking phases were completed in October 2015 (post-planting) and April/May 2016 (post-harvest). The tracking data is integrated into the post-planting and post-harvest structure, even though the data were actually collected in the tracking phase. The questionnaires implemented for tracking households were identical to those used in the main phase of the interview^2^
Data source and location	The LSMS-ISA data for Nigeria covers 500 Enumeration Areas (EAs) sampled from the all the Local Government Areas (LGAs), Six Geopolitical Zones (GPZ), and the 36 States and FCT, Abuja in Nigeria. It also covers the urban and rural areas in each of the States.
Data accessibility	The LSMS-ISA for the three Waves (Wave 1, 2011/2012 sessions; Wave 2, 2013/2014 session; and Wave 3, 2015/2016 session) is available at http://microdata.worldbank.org/index.php/catalog/2734
Related research article	Osabuohien [3]. Large-Scale Agricultural Land Investments and Local Institutions in Africa: The Nigerian Case. Land Use Policy, 39, 155–159.
	Osabohien and Osuagwu [2]. Social Protection Policies and Agricultural Output in Nigeria: Empirical Investigation Using Household Survey Data. Presented at the 4^th^ Covenant University International Conference on E-Governance in Nigeria (CUCEN), Covenant University, Ota, Nigeria, 7–9 May 2017.

**Value of the data**

• LSMS-ISA data is an integration of longitudinal panel survey into GHS that makes it possible to produce a more comprehensive analysis of poverty indicators on socio-economic characteristics for rural households^3^
• LSMS-ISA data helps in building capacity and the development of sustainable systems for the production of accurate and timely information on agricultural households
• LSMS-ISA helps the development of an innovating model for collecting agricultural data
• LSMS generates high-quality data, improving survey methods, and building capacity. The goal of the LSMS is to facilitate the use of household survey data for evidence-based policymaking.

## 1 The data

The rural households’ survey is important source of development data, but in low- and middle-income countries, the capacity to conduct and analyse them widely differs. The LSMS-ISA helps address these issues with respect to agricultural data [1].

The Living Standards Measurement Study-Integrated Surveys on Agriculture (LSMS-ISA), General Household Survey (GHS) Panel is part of a larger, regional project in Sub-Saharan Africa (SSA) to improve agricultural data for analysis. Ethiopia, Mali, Malawi, Niger, Nigeria, Tanzania and Uganda are the seven countries being supported by the World Bank through funding from the Bill and Melinda Gates Foundation (BMGF), to strengthen the production of household-level data on agriculture. This regional project, the LSMS-ISA has the aim of enhancing people׳s understanding of agriculture in SSA, specifically, its role on household welfare, access to agricultural land and poverty reduction [1].

## 2 Experimental design, materials, and methods

The survey instruments for this data set is based on LSMS-ISA for the Household Survey Panel sample which comprises of 5000 households (for three Waves; wave 1, 2011/2012 sessions; Wave 2, 2013/2014 session; and Wave 3, 2015/2016 session) were considered to be characteristics at the zonal and national levels which is made up of both the urban and rural areas.

Wave 3 GHS-Panel comprises three questionnaires^4^ for the two different visits (post planting and post-harvesting seasons). Questionnaire for the survey was given to household members in the survey sample for the visits. The survey was conducted on three different levels "survey on Households, a survey on agriculture and survey on the community". The survey on agriculture involves questionnaire which was given to individuals who are farmers and engages in agricultural activities like crop farming, rearing of livestock and other similar activities. The survey for the community involves questionnaire which was administered to the community in the collection of the necessary information on their socio-economic indicators of the enumeration areas where the sample households live. The questionnaires are presented are summarised herein.

### 2.1 GHS-Panel Household Questionnaire

The Household Questionnaire gives data on socioeconomics; instruction; wellbeing (counting anthropometric estimation for kids and kid vaccination); work and work information accumulation alternatives; nourishment and non-sustenance use; family unit nonfarm pay producing exercises; nourishment security and stuns; security nets; lodging conditions; resources; data and correspondence innovation; and different wellsprings of family unit salary.

### 2.2 GHS-Panel Agriculture Questionnaire

The Agriculture Questionnaire solicits information on the ownership of land and its uses such as farm labour; inputs use; GPS measuring area of land and management of household farmland; machinery used for farming; irrigation; harvesting and crop utilisation; animal holdings and fishing (Table 1, Table 2).

**Table 1:** Household Welfare Questionnaire.

Section	Topic	Respondent
Cover	Cover	Filled by the surveyor
1	Roster	Completed by the head^a^ of HOUSEHOLD or spouse.
2	Education	All individuals for themselves unless under age 12; then information is collected from parent or guardian
3	Labour	All individuals for themselves unless under age 12; then collect the information from parent or guardian
4A	Health	All individuals
4B	Child Development	Children 2–18 years
5	Remittance	All individuals 10 years and above
6	Behaviour	All individuals 10 years and above
6B	Attitude	All individuals 10 years and above
9	Non-Farm Enterprises and Income Generating Activities	Owner or manager of enterprise
10A	Meals Away from Home	Female in the household responsible for food preparation and/or food purchases
10B	Food Consumption and Expenditure	Female in the household responsible for food preparation and/or food purchases
10C	Aggregate Food Consumption	Female in the household responsible for food preparation and/or food purchases
11	Non-Food Expenditures	Most knowledgeable person or person, who is responsible for household purchases
12	Food Security	HOUSEHOLD head or eligible adult
13	Other Household Income	HOUSEHOLD head or eligible adult
14	Safety Nets	HOUSEHOLD head or eligible adult
15A	Economic Shocks and Death	HOUSEHOLD head or eligible adult
15B	Death in the Household	HOUSEHOLD head or eligible adult
15C	Conflict	HOUSEHOLD head or eligible adult
16	Contact Information	

The questionnaires can be obtained from http://surveys.worldbank.org/lsms/integrated-surveys-agriculture-ISA/nigeria#bootstrap-panel--2.

a A person defined as such for the purpose of the survey, irrespective of reason (the oldest by age, decision maker in the household, a person who earns the most income, based on tradition, etc.).

**Table 2:** Household Agricultural Questionnaire.

Section	Topic	Respondent
	Cover	Completed by the surveyor. Household identification (HHID) was copied from HOUSEHOLD to Agriculture Questionnaire
A1	Land	Farmer, owner or manager of plot
A2	Labour	Farmer, owner or manager of plot
11C1	Input cost	Farmer, owner or manager of plot
11D	Fertilizer Acquisition	Farmer, owner or manager of plot
A3i. & ii	Agricultural Production Harvest of Field and Tree crops and crop Disposition	Farmer, owner or manager of plot
A4	Agricultural capital	Farmer, owner or manager of plot
A5	Extension Services	Farmer, owner or manager of plot
A8	Other Agricultural Income: Agricultural By-Product	Farmer or caretaker of animals
A9 (A and B)	Fishing Capital and Revenue	Owner of fishing operations
A10	Network Roster	Farmer, owner or manager of plot

Table 3 shows the details of Waves sample. The Wave 3 sample size for households interviewed in both post-planting and post-harvest visit is 4581. This size is only 135 households less than Wave 2. However, there were some households that were not interviewed in Wave 2 that were found and interviewed in Wave 3.

**Table 3:** Details of GHS-Panel Sample in each Wave.

	Wave 1			Wave 2			Wave 3		
Geo-political Regions	All	Urban	Rural	All	Urban	Rural	All	Urban	Rural
North Central	794	217	577	784	214	570	777	210	567
North East	979	138	659	741	117	624	643	106	537
North West	898	170	728	878	156	722	882	163	719
South East	794	204	590	763	197	566	755	193	562
South-South	769	229	540	761	219	542	744	221	523
South West	864	611	253	789	562	227	780	556	224
**Total**	**4916**	**1569**	**3347**	**4716**	**1465**	**3251**	**4581**	**1449**	**3132**

## Footnotes

4 There are two types of questions used in the questionnaire: i. Closed ended questions: (a) both question text and question code are read: For this type of question, the interviewer must literally read questions and slowly, one-by-one, list of offered codes. In such questions, both question and code are printed in small letters. (b) Only Question Text is Read-: For this type of question, the interviewer reads only the text of the question, waits for the answer and then selects corresponding code and enters it. In this type of question, question text is printed in small letters and question codes are printed in CAPITAL letters. ii. Open ended Questions: For this type of question, the interviewer reads only question text and then enters answer exactly as given by the respondent. For such questions there are no offered answers, and the interviewer enters either words or numbers depending on the question and answer. “Respondent׳s name” is an example of open question where the interviewer enters words.

## Footnotes

1 A part of population representing the whole population. Sample selection is a subject of statistical methods that takes into consideration the characteristics of both the population and individual members of the population, while population is a Set of elements which make the whole, which could be all the people in a country or an entity, all households, etc.

2 Interview is referred to as a procedure by which information on certain person is collected directly from the person. The person giving information on him/her is a “direct respondent”.

3 Household is a Social unit consisting of one or more persons, who use joint accommodation and food. In other words, a household is a group of persons, who normally live in the same household unit (“live under the same roof”), who are or are not related and who eat together (“eat from the same pot”).
