# Supplementary material for: Contributing to agricultural mix:analysis of the living standard measurement study – Integrated survey on agriculture data set
Source: Data Brief. 2018 Jul 27;20:96–100. doi: 10.1016/j.dib.2018.07.057 (PMC6082989; doi:10.1016/j.dib.2018.07.057)

AUTHOR DECLARATION

The author wishes to confirm that there are no known conflicts of interest associated with this publication and there has been no significant financial support for this work that could have influenced its outcome.

I confirm that the manuscript has been read and approved and that there are no other persons who satisfied the criteria for authorship but are not listed. I further confirm that I am the sole author of the manuscript which has been approved and submitted for publication

I confirm that I have given due consideration to the protection of intellectual property associated with this work and that there are no impediments to publication, including the timing of publication, with respect to intellectual property. In so doing, it is confirmed that I have followed the regulations of my institution’s concerning intellectual property.

I understand that the Corresponding Author is the sole contact for the Editorial process (including Editorial Manager and direct communications with the office). I this wise, I am responsible for communicating with editors about the progress, submissions of revisions and final approval of proofs. I confirm that I have provided a current, correct email address which is accessible by the Corresponding Author and which has been configured to accept email from

Thank You,

Romanus Osabohien,

Department of Economics and Development Studies, Covenant University, Ota, Nigeria

[*romanus.Osabohien@covenantuniversity.edu.ng*](mailto:romanus.Osabohien@covenantuniversity.edu.ng)

*18/06/2018*


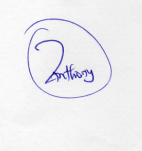

Supplement: Supplementary file 1 — Supplementary material [file mmc1.docx]
